# Rupture of the Perineum and Recto-Vaginal Septum during First Coition

**Published:** 1890-07

**Authors:** Roman L. Sinaisky

**Affiliations:** Slutzk, Russia


### Selections and Abstracts

### RUPTURE OF THE PERINEUM AND RECTO-VAGINAL SEPTUM DURING FIRST COITION.

BY DR. ROMAN L. SINAISKY (Slutzk, Russia.)

A previously quite healthy, newly-married, young Hebrew woman, æt. 23 years, of middling size and make, applied to the author on account of pain on walking and defecation, which symptom had appeared after her first marital intercourse two days previously. She added that the coition had given rise to an excruciating local pain and profuse bleeding, causing her to faint. The examination showed that the woman's external genitals were developed quite normally, and that the hymen (of a semi-lunar variety and a moderate thickness) was intact. On separation of the major labia, the posterior commissure proved to be lacerated, the wound forming a funnel-shaped cavity admitting freely two or three fingers and communicating with the rectum just above the anal sphincter; the vagina contained fæcal gases and matter. There was also present a total rupture of the perineum running along the raphe, but involving only the skin and sub-cutaneous cellular tissues. An operative treatment was proposed but declined by the patient. Dr. Sinaisky discusses at length the question concerning the etiology of the severe lesions found in his patient. He does not entertain any doubt whatever that they were actually contracted during the coition. The patient's husband proved to be a robust young man, æt. 23 years, possessing a large-sized member, but no knowledge concerning marital business (at least he stated that he had never yet had intercouse with women until the present occasion). Since the couple most emphatically declared that neither of them had introduced finger or any foreign body into the woman's genital tract, and since all their statements generally seemed to be altogether trustworthy, the author arrives at the conclusion that the lesions were inflicted solely by some violent and wrongly directed pressure of the inexperienced young man's powerful and stifly erect penis against the base of the hymen, the woman, possibly, lying in some inappropriate posture. The violence might be intensified by virtue of the attempt at coition being undertaken with retracted prepuce (Masalitinoff, Harris, Boriakovsky) the young man being a circumcised Hebrew.

Reviewing the literature of the subject, the author points to the following instances of similarly severe injuries to female genitals during coition: Albert's case ("Hoffman's Handbook of Forensic Medicine"), referring to an Arabian girl, æt. 11 years, in whom the first intercourse with her husband, a robust lad of 16, caused the rupture of the posterior commissure, navacular fossa and vaginal fornix, the latter communicating with the abdominal cavity. 2. Toulmouche's (*ibid*) of rupture of the perineum in a ravished girl, æt. 25 years. 3. Zeiss's (*Centrabalblatt fuer Gynæcologie*, No. 8, 1886) of laceration of the vaginal roof during coition performed in an elbow-knee posture about six weeks after a forceps labor. 4. Chadwick's (*Boston Med. and Surg. Jour.*, April 30, 1885) of rupture of the vagina in a sterile woman æt. 48 years. 5. A. G. Masalitinoff's (*London Medical Record*, May, 1886, p. 214) case of rupture of the perineum in a weak chlorotic Hebrew woman æt. 24, the lesion taking place during the first coitus with her athletic husband who performed the act in a drunken state. 6. Masalitinoff's case (*ibid*) of vesico-vaginal fistula occurring in a Georgian woman æt. 18 years, during her first coition with her husband. 7. Afanasy N. Boiakovsky's (*Vratch*, Nos. 46 and 47, 1886, p. 821) case of rupture of the perineum and vulvo-rectal fistula in a peasant woman, æt. 17 years, who had undersized external genitals, abnormally short genital slit (the distance between the urethral orifice aad posterior commissure being not more than 2 cm.), a deep depression (*cul dc sac*) on the posterior periphery of the vestibule, a narrow pelvic arch (the angle being only 62o against the standard 90o-100o), and a subnormal inclination of the pelvis (40o against the standard 60o). The patient's husband was a robust peasant, of 24 years, with a large sized penis and a retracted foreskin. The first coition was exceedingly painful. On the next morning fecal gases, and on the third day fecal matter began to escape from the woman's genitals. The fistula was successfully closed by Prof. G. E. Rein about 3 years later. Dr. Linoisky's collection may be supplemented by the following cases. W. A. Esipoff's case (*London Medical Record*, May, 1886, p. 214) of rupture of the urethra occurring during the first coition in a young woman æt. 19 years, with imperforate hymen and 2,000 cub. em. blood pent up in the vagina and womb. 2. J. Price's (*The American Obstetric Gazette* May, 1886) of vulvo-rectal fistula arising during the first coitus in a woman, æt. 22 years. 3. Duguer's (*Vratch*, No. 47, p. 843, 1886), of rupture of the perineum and vagina during the first intercourse with the husband possessing a big penis and performing the act in a very rough and violent manner. 4. Blumenthal's (quoted by Price, *i. c.*,) of vulvo rectal fistula, operated by Spencer Wells in 1860. 5 and 6. Diemerbroick's cases (*Anatomia Corporis Humani*, quoted by Boriakovsky, *i. c.*) of rupture of the vagina referring to two newly married young Dutch women, both of whom died from acute anæmia caused by hæmorrhage. 7. Liman's case ("Hoffman's Handbook") of rupture of the perineum.—*Russkaia Meditzina*, No. 46, p. 711, 1889).

VALERIUS IDELSON, (Berne.)

*Annals of Surgery.*

